# Antimicrobial Resistance in *Pasteurellaceae* Isolates from Pyrenean Chamois (*Rupicapra pyrenaica*) and Domestic Sheep in an Alpine Ecosystem

**DOI:** 10.3390/ani11061686

**Published:** 2021-06-05

**Authors:** Irene Torres-Blas, Xavier Fernández Aguilar, Oscar Cabezón, Virginia Aragon, Lourdes Migura-García

**Affiliations:** 1 Departament de Medicina i Cirurgia Animals, Universitat Autònoma de Barcelona, 08193 Bellaterra, Spain; ireto3@gmail.com (I.T.-B.); xfdezaguilar@gmail.com (X.F.A.); Oscar.Cabezon@uab.cat (O.C.); 2 Research and Conservation Department, Zoo de Barcelona, Parc de la Ciutadella s/n, 08003 Barcelona, Spain; 3 Department of Ecosystem & Public Health, University of Calgary, 3330 Hospital Drive NW, Calgary, AB T2N 4N1, Canada; 4 UAB, Centre de Recerca en Sanitat Animal (CReSA, IRTA-UAB), Campus de la Universitat Autònoma de Barcelona, 08193 Bellaterra, Spain; 5 IRTA, Centre de Recerca en Sanidad Animal (CReSA, IRTA-UAB), Campus de la Universitat Autònoma de Barcelona, 08193 Bellaterra, Spain; virginia.aragon@irta.cat

**Keywords:** antimicrobial resistance, chamois, wildlife, *Pasteurellaceae*, *Rupicapra pyrenaica*, wildlife–livestock interface

## Abstract

**Simple Summary:**

Antimicrobial resistance (AMR) is a public health concern. Increasing resistance has arisen due to the inappropriate use of antimicrobial drugs in both, human and veterinary medicine. Although AMR is mainly linked to direct and indirect human activities, little is known about the presence and impact that AMR has on wild animals, especially for those bound to habitats subjected to little human pressure. Our study analyzed the AMR profiles of different *Pasteurellaceae* species obtained from the infected lungs of Pyrenean chamois (*Rupicapra pyrenaica*) and domestic sheep found in a National Hunting Reserve from Spain. We have found evidence of the presence of AMR in both animal species. Furthermore, some isolates were resistant to cephalosporins and fluoroquinolones, which are antimicrobials of critical importance in human medicine. Further research is needed to discern pathways of AMR dissemination within natural environments and assess its persistence in wildlife in order to prevent further AMR spreading at a global scale.

**Abstract:**

Antimicrobial resistance (AMR) has spread worldwide due to the inappropriate use of antimicrobial drugs in human and veterinary medicine, becoming a public health problem. However, little is known about its occurrence and maintenance in wild animals, and very few studies have been carried out in ecosystems subjected to low human pressure. In our study, nasal and lung swabs were collected from hunted Pyrenean chamois (*Rupicapra pyrenaica*), and nasal swabs from sympatric domestic sheep were also collected. The swabs were cultured in agar plates to obtain bacterial isolates from the *Pasteurellaceae* family. The presence of AMR was assessed in a total of 28 *Pasteurellaceae* isolates from 45 Pyrenean chamois, and 9 isolates from sympatric domestic sheep found in the National Hunting Reserve of Freser-Setcases (Northeastern Pyrenees, Spain). The isolates belonged to one of the following three species: *Pasteurella multocida, Mannheimia haemolytica* and *Bibersteinia trehalosi.* Some *P. multocida* and *M. haemolytica* isolates tested positive for AMR. The statistical analysis revealed no differences between the AMR levels from chamois and domestic sheep isolates. However, one *P. multocida* of chamois origin presented resistance to cephalosporins and fluoroquinolones, which are antibiotics of critical importance for human health. Further studies are required to elucidate potential routes of dissemination of AMR genes in natural environments and assess any significant persistence in wildlife to design risk mitigation actions.

## 1. Introduction

Antimicrobial resistance (AMR) is a natural phenomenon; most antimicrobial agents are produced by free-living fungi, plants and bacterial species that are present in all environments, enabling them to fight infections and directly compete for ecological niches [1]. The genes conferring resistance to antimicrobial drugs are positively selected in microbial populations under the environmental pressure of these compounds [2]. The excessive and inappropriate use of antimicrobial agents by humans has led to the selection, emergence and spread of multidrug resistance in both pathogenic and commensal bacteria [3,4]. Moreover, antimicrobial drugs can persist in soil and aquatic environments, exercising also selective pressure over bacteria in these environments [5,6].

The emergence of AMR in both Gram-positive and Gram-negative bacteria has resulted in difficult-to-treat or untreatable infections, an increase in morbidity and mortality, and an increase in the costs of treating infectious diseases [7,8]. These factors have turned AMR into one of the main public health concerns worldwide [9,10]. Overall, the World Health Organization (WHO) has foreseen the beginning of a post-antimicrobial era [11].

Despite the importance of AMR, its occurrence and maintenance in wild animals is poorly understood. Antibiotic-resistant bacteria have already been described in different species [12,13]. AMR in wildlife can be a public health concern if associated with zoonotic pathogens that are transmissible from wildlife to humans, or vice versa. Wildlife may also provide a mechanism for the spread and maintenance of resistance genes into natural systems [3], and for predicting the emergence of resistant pathogens [9]. Therefore, wildlife has been proposed as a good sentinel to locally monitor and predict the emergence of AMR [9].

The study of AMR occurrence in remote habitats, or in ecosystems subjected to limited human influence, can help to identify levels of AMR in natural environments and assess how anthropogenic activities are influencing AMR in bacterial populations worldwide [14,15]. There are a few studies carried out in such isolated environments, for instance the Antarctica [15,16,17] or in alpine ecosystems [18].

Up to date, most of the studies on AMR and wildlife have focused on Enterococci [9], *Enterobacteriaceae* [19,20], *Campylobacter* spp., *Listeria monocytogenes* [20], or even methicillin-resistant *Staphylococcus aureus* [21]. Yet, very few studies have focused on the bacteria involved in respiratory infections, such as those belonging to the family *Pasteurellaceae*.

The aim of this study was to assess the antimicrobial susceptibility profile of the bacteria involved in respiratory infections of the *Pasteurellaceae* family, isolated from sympatric Pyrenean chamois (*Rupicapra pyrenaica*) and domestic sheep that graze in alpine meadows. These studies are vital to establish baseline data of AMR occurrence and assess their extent in an area with a priori low human influence.

## 2. Materials and Methods

### 2.1. Study Area

The present study was performed in the Freser-Setcases National Game Reserve (FSNGR) in the Pyrenees, Northeastern Spain. This area is a high mountain habitat with strong seasonal influence and is mainly composed of alpine and subalpine ecosystems. Pyrenean chamois is the most abundant ungulate species, although mouflon (*Ovies aries musimon*), roe deer (*Capreolus capreolus*), red deer (*Cervus elaphus*) and wild boar (*Sus scrofa*) also occur in much lower numbers. Domestic ruminants, including domestic sheep, cattle and horses, also dwell with the wild ungulates during the grazing period from May to November [22].

### 2.2. Sample Collection and Processing

Between 2015 and 2017, a total of 37 *Pasteurellaceae* isolates were obtained from pneumonic lesions in Pyrenean chamois (*n* = 45) legally hunted in FSNGR during the regular hunting season (August–November), and from nasal swabs of domestic sheep (*n* = 24) in two flocks that seasonally graze at FSNGR.

For each sampled Pyrenean chamois, both nasal and lung swabs with Amies transport medium were collected. In the case of domestic sheep, a single nasal swab with Amies transport medium was also collected. All swabs were carefully labeled and stored at 4 °C and processed in the lab within the first 24 h post-collection. Amies swabs were cultured on Polivytex agar plates (Biomerieux, Madrid, Spain) and incubated for 48 h at 37 °C and 5% CO_2_ [23]. Macroscopic morphologically compatible colonies with *Pasteurellaceae* were replated to obtain a pure culture and were further identified using a 16S rRNA PCR, following previously described protocols [24]. The positive controls used for the PCR were from a *P. multocida* isolate Sanger-sequenced from our strain collection. Isolates that were confirmed to belong to the family *Pasteurellaceae* were then stored in 20% glycerol/brain heart infusion (BHI; Oxoid) at −80 °C until further analysis.

### 2.3. Minimum Inhibitory Concentration (MIC)

MIC was determined by microbroth dilution method (Thermo Scientific^TM^ Sensititre^TM^, Waltham, MA, USA) following the Clinical Laboratory Standards Institute guidelines (CLSI, Wayne, PA, USA, 2018). Antimicrobials and concentrations tested were as follows: chlortetracycline (0.5–8 μg/mL), oxytetracycline (0.5–8 μg/mL), gentamicin (1–16 μg/mL), spectinomycin (8–64 μg/mL), neomycin (4–32 μg/mL), florfenicol (0.25–8 μg/mL), penicillin (0.12–8 μg/mL), ampicillin (0.25–16 μg/mL), ceftiofur (0.25–8 μg/mL), danofloxacin (0.12–1 μg/mL), enrofloxacin (0.12–2 μg/mL), sulphadimethoxine (256 μg/mL), trimethoprim/sulfamethoxazole (2/38 μg/mL), tylosin tartrate (0.5–4 μg/mL), tulathromycin (1–64 μg/mL), tilmicosin (4–64 μg/mL), clindamycin (0.25–16 μg/mL) and tiamulin (1–32 μg/mL). MIC determinations and clinical breakpoints were used in accordance with criteria provided by the CLSI (CLSI, Wayne, PA, USA, 2018). Multidrug resistance was defined as resistance to three or more families of antimicrobials [25].

### 2.4. Statistical Analysis

Fisher’s exact tests were applied in order to evaluate statistical differences between resistance found in bacterial species, and whether resistance was related to animal species. Pairwise Test of Independence for nominal data was applied as a post-hoc test. The *p*-values were considered statistically significant when below 0.05 (*p* < 0.05). *Bibersteinia trehalosi* was not included on the statistical analysis due to a lack of representative samples and absence of data regarding MIC breakpoints. For the statistical analysis, an isolate was considered resistant when it presented resistance to at least 1 antimicrobial family. Factors included in the statistical model were animal species, bacteria species, and resistance profile. The statistical analysis was performed using the R 3.3.3 program [26].

## 3. Results

In total, 20 *Pasteurella multocida* (19 from chamois and 1 from sheep), 14 *Mannheimia haemolytica* (6 from chamois and 8 from sheep) and three *Bibersteinia trehalosi* from chamois were isolated (Table 1).

Among the *P. multocida* isolates in Pyrenean chamois (*n* = 19), 10.5% were resistant to ampicillin, 10.5% to penicillin and 5.2% to ceftiofur. Additionally, intermediate resistance was detected for enrofloxacin (5.3%), spectinomycin (5.36%) and florfenicol (5.3%) (Table 2, MIC values in Supplementary Table S1). One *P. multocida* of chamois origin was multidrug resistant, exhibiting resistance to beta-lactams, fluoroquinolones and phenicols. All of the *M. haemolytica* isolates of chamois (*n* = 6) were pansusceptible (Table 3, MIC values in Supplementary Table S2). Neither the clinical breakpoints nor epidemiological cut-off values have been described for *B. trehalosi* by CLSI or the European Committee on Antimicrobial Susceptibility Testing (EUCAST), yet the MIC results obtained for our isolates belonging to this species are displayed on Supplementary Table S3.

The statistical analysis revealed no significant differences in the AMR profiles between bacterial species or animal species (chamois and sheep).

## 4. Discussion

Monitoring AMR in geographic areas with a relatively low level of human influence provides a suitable system for studying AMR in natural habitats. Surprisingly, the isolates from *Pasteurellaceae* obtained from chamois exhibited similar levels of resistance to several antimicrobial agents used in human and animal health, such as beta-lactams and macrolides. Furthermore, this is the first report of resistance to a third-generation cephalosporin (ceftiofur) in a *P. multocida* isolate of wildlife origin. The bacteria from the *Pasteurellaceae* family can acquire resistance to antimicrobials by mutations or horizontal gene transfer from different sources, including other bacteria species. In this regard, mobile genetic elements, such as plasmids or transposons, are the main elements that contribute to the dissemination of AMR, and *Pasteurellaceae* can act as both receptors and donors of these genetic elements [27]. In fact, resistance to cephalosporin is usually determined by the presence of extended-spectrum beta-lactamases (ESBLs). It is difficult to elucidate the origin of this resistance phenotype since these chamois are not subjected to an AMR-selective environment. Recent studies have found that AMR is a multifactorial problem, with intrinsic links between the human, animal and environmental interface. It has been demonstrated that interactions between those three settings allow the movement not only of bacteria, but also of antimicrobial resistance genes (ARGs). Environmental factors, such as water and soil, can serve as pools of ARGs. Additionally, given the high use of antibiotics in farm animals in Europe, the sympatric sheep could have acted as carriers of AGRs [28]. However, it is worth pointing out that most of the studies in the literature are focused on *Enterobacteriaceae*, rather than respiratory-related bacteria. In general, AMR levels in *Pasteurellaceae* from domestic animal species have shown to be low but with an increasing tendency [27,29].

In our case, interactions between livestock and chamois may occasionally occur in alpine meadows [30], and seasonal AMR transmission between domestic and wild ungulates cannot be excluded. The potential sources of interaction between the Pyrenean chamois and domestic sheep could include the supplementation of salt or even the presence of domestic sheep feces within the pastures. It is also unclear the role that the environment plays in our scenario as a potential reservoir for ARGs, as it has been proposed for *Enterobacteriaceae*. However, there are a lack of studies based in other species and further work should be designed to unravel possible ARGs transmission routes between respiratory bacteria and the environmental pool of ARGs.

The most common antibiotics used to treat respiratory infections in domestic ruminants include ceftiofur, tulathromycin (macrolide), sulfonamides, tetracyclines, streptomycin and florfenicol [29,31]. The finding of a cephalosporin-resistant isolate is interesting since third and fourth-generation cephalosporins are considered “critically important” antibiotics in human medicine [32,33]. Furthermore, resistance to ceftiofur has been described in recent years in *P. multocida* from cattle origin [34]. *P. multocida* isolates analyzed herein exhibited similar AMR profiles to those described from cattle in Europe [30]; in general, a low level of resistance to all antimicrobials with increasing resistance to florfenicol and spectinomycin. El Garch et al. (2016) [35] also reported susceptibility to florfenicol and increased resistance to spectinomycin in cattle.

Studies focusing on *P. multocida* and *M. haemolytica* described rising numbers of AMR worldwide in food-producing animals [30]. In agreement with our data, *M. haemolytica* typically exhibited more resistance than *P. multocida*. Although our results did not show statistical significance regarding resistance profiles between *M. haemolyitica* isolated from chamois and sheep, resistance to danofloxacin and tulathromycin was detected in sheep isolates, probably as a result of antimicrobial drug usage during rearing.

Finally, the number of isolates of *B. trehalosi* obtained in this study is too small to get to any conclusive results. However, considering the lack of information in the literature regarding AMR for this bacterial species and the lack of clinical breakpoints, the results from this study contribute as valuable reference for further studies.

## 5. Conclusions

In conclusion, AMR levels found in the *Pasteurellaceae* isolates from wild and domestic animals in an alpine ecosystem from Northeastern Spain are consistent with that described in other livestock from Europe. The similar frequency of AMR detection in isolates from chamois may indicate an indirect anthropogenic influence for the AMR spread in these ecosystems. Furthermore, we report the presence of cephalosporin and fluoroquinolone resistant isolates, which are antimicrobials of clinical importance for human health. Further studies are needed to elucidate potential routes of dissemination of AMR genes in natural environments and assess any significant persistence in wildlife in order to implement risk mitigation actions.

## Figures and Tables

**Table 1:** Number of antimicrobial resistance (AMR) detected divided by bacteria species and animal species. We considered that AMR was present if an isolate showed intermediate or total resistance to a specific antimicrobial family, following MIC criteria from CLSI guidelines. Considered antibiotics are ceftiofur, penicillin, ampicillin, danofloxacin, enrofloxacin, tulathromycin, tilmicosin, chlortetracycline, oxytetracycline, spectinomycin and florfenicol.

Bacteria	Chamois		Sheep		All	
	Analyzed	AMR *	Analyzed	AMR	Analyzed	AMR
*Pasteurella multocida*	19	7	1	0	20	7
*Mannheimia haemolytica*	6	1	8	5	14	6
*Bibersteinia trehalosi*	3	-	-	-	-	-
Total	28	8	9	5	37	13

* Minimum inhibitory concentration (µg/mL), established by the CLSI guidelines: ceftiofur: ≥8 _Pm_/≥8 _Mh_, penicillin: ≥1 _Pm_/≥1 _Mh_, ampicillin: ≥0.25 _Pm_/≥0.25 _Mh_, danofloxacin: ≥1 _Pm_/≥1 _Mh_, enrofloxacin: ≥2 _Pm_/≥2 _Mh_, tulathromycin: ≥64 _Pm_/≥64 _Mh_, tilmicosin: ≥32 _Pm_/≥32 _Mh_, chlortetracycline: ≥8 _Pm_/≥8 _Mh_, oxytetracycline: ≥8 _Pm_/≥8 _Mh_, spectinomycin: ≥128 _Pm_ /≥128 _Mh_ and florfenicol: ≥8 _Pm_/≥8 _Mh_. X_Pm_: MIC value for *Pasteurella multocida;* X_Mh_: MIC value for *Mannheimia haemolytica*.

**Table 2:** Number of *Pasteurella multocida* isolates found in chamois and domestic sheep, classified as susceptible (S), intermediate (I) or fully resistant (R) to the antibiotics tested. We considered that AMR was present if an isolate showed intermediate or total resistance to a specific antimicrobial family, following MIC criteria from CLSI guidelines. Considered antibiotics are ceftiofur, penicillin, ampicillin, danofloxacin, enrofloxacin, tulathromycin, tilmicosin, chlortetracycline, oxytetracycline, spectinomycin and florfenicol.

*P. multocida. N* = 20 (RP = 19, OA = 1)					
Antimicrobial Family	Antimicrobial Agent	Species	S (%)	I (%)	R (%)
Cephalosporins	Ceftiour	RP	94.7	0	5.3
		OA	100	0	0
Penicillins	Penicillin	RP	89.47	0	10.53
		OA	100	0	0
	Ampicillin	RP	89.57	0	10.5
		OA	100	0	0
Fluoroquinolones	Danofloxacin	RP	100	0	0
		OA	100	0	0
	Enrofloxacin	RP	94.74	5.3	0
		OA	100	0	0
Macrolides	Tulathromycin	RP	100	0	0
		OA	100	0	0
	Tilmicosin	RP	100	0	0
		OA	100	0	0
	Tylosin tartrate	RP	-	-	-
		OA	-	-	-
Tetracyclines	Chlortetracycline	RP	100	0	0
		OA	100	0	0
	Oxytetracycline	RP	100	0	0
		OA	100	0	0
Aminoglycosides	Gentamicin	RP	-	-	-
		OA	-	-	-
	Neomcyin	RP	-	-	-
		OA	-	-	-
	Spectinomycin	RP	-	-	-
		OA	-	-	-
Fenicols	Florfenicol	RP	94.7	5.3	0
		OA	100	0	0
Sulphonamides	Sulphadimetoxine	RP	-	-	-
		OA	-	-	-
	Trimetroprim/Sulfametoxazole	RP	-	-	-
		OA	-	-	-
Lincosamides	Clindamycin	RP	-	-	-
		OA	-	-	-
	Tiamulin	RP	-	-	-
		OA	-	-	-

N: sample size. RP: *Rupicapra pyrenaica* (chamois); OA: *Ovis aries* (domestic sheep). NA: not calculated due to the absence of CLSI established breakpoints.

**Table 3:** Number of *Mannheimia haemolytica* isolates found in chamois and domestic sheep, classified as susceptible (S), intermediate (I) or fully resistant (R) to the antibiotics tested. We considered that AMR was present if an isolate showed intermediate or total resistance to a specific antimicrobial family, following MIC criteria from CLSI guidelines. Considered antibiotics are ceftiofur, penicillin, ampicillin, danofloxacin, enrofloxacin, tulathromycin, tilmicosin, chlortetracycline, oxytetracycline, spectinomycin and florfenicol.

*M. haemolytica N* = 14 (RP = 6, OA = 8)					
Antimicrobial Family	Antimicrobial Agent	Species	S (%)	I (%)	R (%)
Cephalosporins	Ceftiour	RP	100	0	0
		OA	100	0	0
Penicillins	Penicillin	RP	100	0	0
		OA	87.50	12.50	0
	Ampicillin	RP	100	0-	0
		OA	100	-	-
Fluoroquinolones	Danofloxacin	RP	100	0	0
		OA	87.50	0	12.50
	Enrofloxacin	RP	100	0	0
		OA	87.50	12.50	0
Macrolides	Tulathromycin	RP	100	0	0
		OA	87.50	0	12.50
	Tilmicosin	RP	100	0	0
		OA	100	0	0
	Tylosin tartrate	RP	-	-	-
		OA	-	-	-
Tetracyclines	Chlortetracycline	RP	100	0	0
		OA	100	0	0
	Oxytetracycline	RP	100	0	0
		OA	100	0	0
Aminoglycosides	Gentamicin	RP	-	-	-
		OA	-	-	-
	Neomcyin	RP	-	-	-
		OA	-	-	-
	Spectinomycin	RP	100	0	0
		OA	100	0	0
Fenicols	Florfenicol	RP	100	0	0
		OA	100	0	0
Sulphonamides	Sulphadimetoxine	RP	-	-	-
		OA	-	-	-
	Trimetroprim/Sulfametoxazole	RP	-	-	-
		OA	-	-	-
Lincosamides	Clindamycin	RP	-	-	-
		OA	-	-	-
	Tiamulin	RP	-	-	-
		OA	-	-	-

N: sample size. RP: *Rupicapra pyrenaica* (chamois); OA: *Ovis aries* (domestic sheep). -: not calculated due to the absence of CLSI established breakpoints.

## Data Availability

All data generated in this study is included in the article. Further information on data and samples is available from the corresponding author on request.

## Supplementary Materials

The following are available online at https://www.mdpi.com/article/10.3390/ani11061686/s1, Table S1: MIC distribution frequencies of *Pasteurella multocida* isolates, Table S2: MIC distribution frequencies of *Mannheimia haemolytica* isolates, Table S3: MIC distribution frequencies of *Biberstenia trehalosi* isolates from Pyrenean chamois.
