# Supplementary material for: Antimicrobial Resistance in Pasteurellaceae Isolates from Pyrenean Chamois (Rupicapra pyrenaica) and Domestic Sheep in an Alpine Ecosystem
Source: Animals (Basel). 2021 Jun 5;11(6):1686. doi: 10.3390/ani11061686 (PMC8226726; doi:10.3390/ani11061686)
Supplement: Supplementary file 1 [file animals-11-01686-s001.zip › animals-1232782-supplementary.pdf]

Supplementary Table S1. MIC distribution frequencies of *Pasteurella multocida* isolates.

ANTIMICROBIAL FAMILY	Antimicrobial agent	Sp.	<0.12	0.12	0.25	0.5	1	2	4	8	16	32	64	128	<2/38	>2/38	<256	>256	S(%)	I(%)	R(%)
			P. multocida (n = 20)																		
			MIC values (µg/mL)																		
CEPHALOSPORINS	Ceftiofur	RP		**17**		**1**					**1**								94.7	0	5.3
		OA		**1**															100	0	0
PENICILLINS	Penicillin	RP	17				1				1								89.47	0	10.53
		OA	1																100	0	0
	Ampicillin	RP		16		1		1			1								89.57	0	10.5
		OA		1															100	0	0
FLUOROQUINOLONES	Danofloxacin	RP	19																100	0	0
		OA	1																100	0	0
	Enrofloxacin	RP	18			1													94.74	5.3	0
		OA	1																100	0	0
MACROLIDES	Tulathromycin	RP				14		2	2	1									100	0	0
		OA				1													100	0	0
	Tilmicosin	RP						19											100	0	0
		OA						1											100	0	0
	Tylosin tartrate	RP							1	13	2	2	1						-	-	-
		OA							1										-	-	-
TETRACYCLINES	Chlortetracycline	RP			16		3												100	0	0
		OA					1												100	0	0
	Oxytetracycline	RP			16		3												100	0	0
		OA			1														100	0	0
AMINOGLYCSIDES	Gentamicin	RP				14	1	3	1										-	-	-
		OA				1													-	-	-
	Neomycin	RP						17		2									-	-	-
		OA						1											-	-	-
	Spectinomycin	RP						16			1	1	1						94.7	5.3	0
		OA						1											100	0	0
FENICOLS	Florfenicol	RP		14	1	3			1										94.7	5.3	0
		OA		1															100	0	0
SULPHONAMIDES	Sulphadimethoxine	RP															8	11	-	-	-
		OA															1		-	-	-
	Trimethoprim/ Sulfametoxazole	RP													13	6			-	-	-
		OA													1				-	-	-
LINCOSAMIDES	Clindamycin	RP							3	11	5								-	-	-
		OA							1										-	-	-
	Tiamulin	RP							1	7	10		1						-	-	-
		OA									1								-	-	-

The value included in each cell corresponds to the number of isolates which tested positive for that MIC value. *Sp.: species; RP: Pyrenean chamois; OA: sheep.* Grey cells indicate a concentration value that was not available on the MIC plate for that antimicrobial agent.Values under the lowest concentration indicate MIC values that are lower than the lowest concentration within the range. Values above the highest concentration indicate MIC values which are higher than the highest concentration within the range. When available, susceptible and resistant breakpoints* are indicated with a simple (susceptible) or double (resistant) lines. Values compressed between both lines indicate MIC values for intermediate resistance. NA: not calculated due to the absence of CLSI established breakpoints.

Supplementary Table S2. MIC distribution frequencies of *Mannheimia haemolytica* isolates.

			M. haemolytica (n = 14)																		
			MIC values (µg/mL)																		
**ANTIMICROBIAL FAMILY**	Antimicrobial agent	Sp.	<0.12	0.12	0.25	0.5	1	2	4	8	16	32	64	128	<2/38	>2/38	<256	>256	S (%)	I (%)	R (%)
**CEPHALOS-PORINS**	Ceftiofur	RP		6															100	0	0
		OA		8															100	0	0
**PENICILLINS**	Penicillin	RP	5		1														100	0	0
		OA	6		1	1													87.50	12.50	0
	Ampicillin	RP		6															100	0	0
		OA		8															100	0	0
**FLUOROQUINOLONES**	Danofloxacin	RP	6																100	0	0
		OA	7				1												87.5	0	12.5
	Enrofloxacin	RP	6																100	0	0
		OA	7				1												87.50	12.50	0
**MACROLIDES**	Tulathromycin	RP						1	4	1									100	0	0
		OA				3		1	2		1		1						87.50	0	12.50
	Tilmicosin	RP						4		2									100	0	0
		OA						8											100	0	0
	Tylosin tartrate	RP										1	5						-	-	-
		OA						3	4			1							-	-	-
**TETRACYCLINES**	Chlortetracycline	RP			3		2	1											100	0	0
		OA			8														100	0	0
	Oxytetracycline	RP			5		1												100	0	0
		OA			8														100	0	0
**AMINOGLYCOSIDES**	Gentamicin	RP				3		2	4										-	-	-
		OA				7		2											-	-	-
	Neomycin	RP						5		1									-	-	-
		OA						8											-	-	-
	Spectinomycin	RP									3	3							100	0	0
		OA									1	7							100	0	0
**FENICOLS**	Florfenicol	RP		1		1	4												100	0	0
		OA		6		1		1											100	0	0
**SULPHONAMIDES**	Sulphadimethoxine	RP															3	3	-	-	-
		OA															7	1	-	-	-
	Trimethoprim/ Sulfametoxazole	RP													5	1			-	-	-
		OA													8				-	-	-
**LINCOSAMiDES**	Clindamycin	RP							4	1	1								-	-	-
		OA					1	2	4				1						-	-	-
	Tiamulin	RP								1	4	1							-	-	-
		OA			1				2	2	2		1						-	-	-

The value included in each cell corresponds to the number of isolates which tested positive for that MIC value. *Sp.: species; RP: chamois; OA: sheep*. Grey cells indicate a concentration value that was not available on the MIC plate for that antimicrobial agent. Values under the lowest concentration indicate MIC values that are lower than the lowest concentration within the range. Values above the highest concentration indicate MIC values which are higher than the highest concentration. When available, susceptible and resistant breakpoints* are indicated with a simple or double lines respectively. Values compressed between both lines indicate the intermediate resistance. NA: not calculated due to the absence of CLSI established breakpoints.

Supplementary Table S3. MIC distribution frequencies of *Biberstenia trehalosi* isolates from Pyrenean chamois.

	B.trehalosi (n = 3)																			
**ANTIMICROBIAL FAMILY**	Antimicrobial Agent	MIC values (μg/mL)																		
		<0.12	0.12	0.25	0.5	1	2	4	8	16	32	64	128	<2/38	>2/38	<256	>256	S (%)	I (%)	R (%)
**CEPHALOSPORINS**	Ceftiofur		3															-	-	-
**PENICILLINS**	Penicillin	1		1		1												-	-	-
	Ampicillin		3															-	-	-
**FLUOROQUINOLONES**	Danofloxacin	1		2														-	-	-
	Enrofloxacin	3																-	-	-
**MACROLIDES**	Tulathromycin				1		2											-	-	-
	Tilmicosin						3											-	-	-
	Tylosin tartrate									1	2							-	-	-
**TETRACYCLINES**	Chlortetracycline			3														-	-	-
	Oxytetracicline			3														-	-	-
**AMINOGLYCOSIDES**	Gentamicin				3													-	-	-
	Neomycin						2		1									-	-	-
	Spectinomycin								1		2							-	-	-
**FENICOLS**	Florfenicol				2	1												-	-	-
**SULPHONAMIDES**	Sulphadimethoxine															3		-	-	-
	Trimethoprim/ sulfamethoxazole													3				-	-	-
**LINCOSAMIDES**	Clindamycin						2	1										-	-	-
	Tiamulin								2	1								-	-	-

The value included in each cell corresponds to the number of isolates which tested positive for that MIC value. Grey cells indicate a concentration value that was not available on the MIC plate for that antimicrobial agent. Dilution ranges tested for each antimicrobial agent are those contained within the white area. Values under the lowest concentration indicate MIC values that are lower than the lowest concentration within the range. Values above the highest concentration indicate MIC values which are higher than the highest concentration within the range. No CLSI established breakpoints are available for *B. trehalosi.*
